# Atypical Location of Diffuse Large B-cell Lymphoma in the Nasal Septum

**DOI:** 10.7759/cureus.28085

**Published:** 2022-08-16

**Authors:** Veshesh Patel, Collin J Tacy, Trevor Creamean, Adiraj Sibia, Jayesh Patel

**Affiliations:** 1 Osteopathic Medicine, Nova Southeastern University Dr. Kiran C. Patel College of Osteopathic Medicine, Fort Lauderdale, USA; 2 Osteopathic Medicine, Nova Southeastern University Dr. Kiran C. Patel College of Osteopathic Medicine, Clearwater, USA; 3 Department of Biology, Nova Southeastern University, Fort Lauderdale, USA; 4 Department of Otolaryngology, McLaren Oakland Hospital, Titusville, USA

**Keywords:** nasal congestion, non hodgkin's lymphoma, chemo radiotherapy (chemo-rt), paranasal sinus diseases, airway disorders, large b cell lymphomas

## Abstract

Diffuse large B-cell lymphoma (DLBCL) is the most prevalent subtype of non-Hodgkin’s lymphoma (NHL). This subtype can be present in various extranodal sites, including the brain, bones, intestines, kidneys, adrenal glands, and other soft tissues. As demonstrated in this case, one rare site of DLBCL is the nasal septum, which presents as a rapidly enlarging mass resistant to antibiotics and steroids. The definitive diagnosis for this case involved biopsy, but further workup, such as computed tomography (CT) and fluorescence in situ hybridization (FISH), helped support the diagnosis of DLBCL. While determining the stage of the lymphoma, treatment with R-CHOP chemotherapy (rituximab, cyclophosphamide, doxorubicin, vincristine, and prednisone) was initiated immediately. This case demonstrates a rare presentation of DLBCL in the nasal septum and describes the significance of urgent examination as well as treatment.

## Introduction

Diffuse large B-cell lymphomas (DLBCL) are the most common lymphoma and are a type of non-Hodgkin’s lymphoma (NHL). It is estimated to have an incidence rate of 150,000 cases annually worldwide [1]. Depending on the primary site of involvement, DLBCL can present with a variety of symptoms, but most individuals can present with "B symptoms," which include fever, weight loss, and night sweats. Representing 30% of all cases of non-Hodgkin’s lymphoma, patients with DLBCL are typically presented as an aggressively growing mass, lymphadenopathy, extranodal disease, or a combination. Diagnosis is typically made through excisional biopsy of the lymph node or fine-needle aspiration, but computed tomography (CT), positron emission tomography (PET), flow cytometry (eg, CD20, CD22, CD30, CD45), and fluorescence in situ hybridization (FISH) can also support the diagnosis and predict prognosis [2]. One rare extranodal site of DLBCL involves the nasal region, such as the nasal septum and paranasal sinuses, which can present with symptoms like epistaxis, sinusitis, headache, and nasal swelling. Nasal involvement of DLBCL is potentially fatal due to the lymphoma's ability to rapidly grow in the respiratory tract, resulting in airway compression, obstruction, or involvement of adjacent structures, requiring urgent treatment. The mainstay treatments for B-cell lymphomas include R-CHOP immunochemotherapy consisting of rituximab, cyclophosphamide, doxorubicin, vincristine, and prednisone. This case details a presentation of DLBCL at a rare extranodal site.

## Case presentation

A 72-year-old female with a past medical history of hypertension and hyperlipidemia presented to the clinic with chronic nasal congestion and sinus pressure. Over the last six weeks, the patient reported no resolution of symptoms with the use of prednisone, azithromycin, levofloxacin, or oxymetazoline nasal sprays. The patient denied any recent surgeries or hospitalizations. She denied any complaints of fever, chills, rigors, nausea, vomiting, or any other constitutional symptoms.

**Figure 1:**
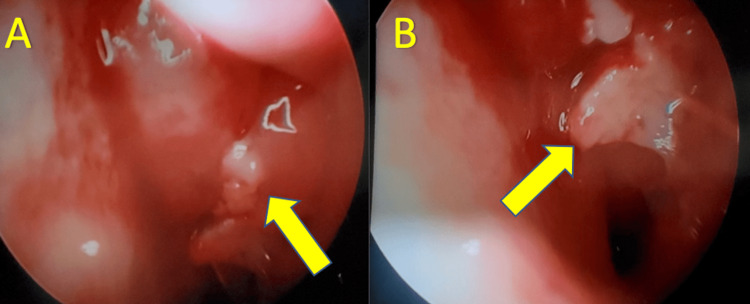
Endoscopic examination of the right and left nasal septum Demonstrates moderate amount of granular, inflamed tissue on the left (A) and right (B) nasal septum, respectively. The left nasal septum (A) illustrates a more extensive and friable mass, with associated bulging.

**Figure 2:**
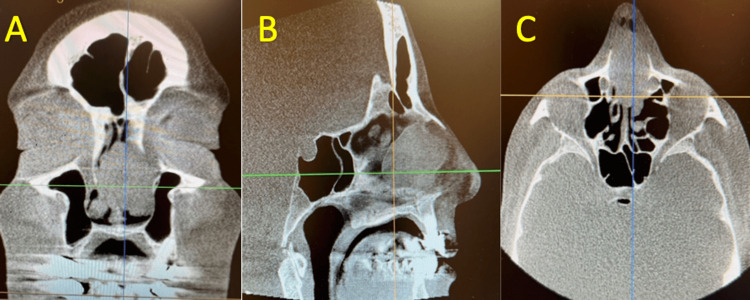
Computerized tomography scan of the nose and sinuses CT imaging reveals a large mass in the right and left nasal septum on coronal (A), sagittal (B), and axial (C) view, respectively. Coronal view (A) depicts a greater left sided nasal septum involvement of the mass compared to the right nasal septum.

After follow-up with an otolaryngologist, local examination with nasal endoscopy showed a mass in the bilateral nasal septum (Figure 1), which was initially attributed as a polyp. On CT of the sinuses, it was reported there were large septal bilateral maxillary sinus masses, as shown in Figure 2. After these concerning findings, a biopsy of the lesion was performed. The preliminary biopsy results were suggestive of a possible B-cell lymphoma. On further workup, FISH analysis of the sampled tissue found there was positive staining for CD20 and BCL2, and partially positive for MYC (60%), BCL6 (40%), MUM1 (40%), and Ki-67 (70%). It was also noted that tissue sections demonstrated a diffuse infiltrate of medium to large-sized cells with vesicular chromatin, conspicuous nucleoli, and scant to moderate amounts of cytoplasm in a background of scattered tingible body macrophages, necrosis, and edema.

Given the findings on CT, biopsy, and FISH, the diagnosis of DLBCL was made for the afebrile patient with antibiotic and steroid-resistant sinusitis. For appropriate staging, the patient was offered further diagnostic options, including a PET scan of the whole body, which noted no other quantitatively significant hypermetabolic abnormalities except in the anterior nares, nasal cavity, and sinonasal cavity. Taking into consideration no distant spread, the final staging was consistent with stage 1A non-bulky lymphoma for this patient. Treatment with three cycles of R-CHOP chemotherapy was initiated, with the option of undergoing radiation following completion. On final follow-up, the patient reported minimal side effects and achieved a positive response to treatment, such as decreased tumor size and alleviation of symptoms.

## Discussion

Multiple studies in the literature have reported T-cell and natural killer (NK) cell lymphomas involving the nasal cavity [3]. However, our case reports nasal septal involvement of a common B-cell lymphoma. Additionally, most NHLs involve the sinuses, without nasal disease, or the Waldeyer’s ring, which is made up of the tonsils, adenoids, and other lymphoid tissue [4]. Although the median age of presentation for a B-cell lymphoma is 50 years and more predominant in males, our older female patient had the most common subtype of NHL in the anterior nasal septum without any constitutional symptoms. A definitive diagnosis was made through biopsy and then supported by characteristic histopathologic features and immunohistochemical markers of the tissue specimen. Due to the infrequency of DLBCL in the nasal septum, this case served to illustrate the unusual nature of this cancer.

Despite the various imaging modalities available to providers, the most appropriate next step in diagnosing this patient's unusual presentation of DLBCL is to gather a thorough history and physical examination. A presentation of a nasal cavity lymphoma can be characterized as an ulcerative, necrotic, or inflamed mass with potential extension to adjacent structures, such as facial, palate, cartilage, and bony structures. On examination with nasal endoscopy, it is likely to see extensive pale, friable, granular tissue with associated pus, crusting, or hemorrhage [5]. These findings can lead to the symptoms found in our patient (e.g., nasal congestion, sinusitis, and nasal pressure). It is imperative to treat the underlying cause of these symptoms to prevent complications such as fistula formation, osseous necrosis, and mucosal ulceration. Our case demonstrated similar clinical manifestations described above, but with no evidence of complications.

The prognosis of the nasal-involved DLBCL is dependent on the extent, staging, and spread of distant structures of the disease. It has been found that two-thirds of patients remain in the remission phase after initial therapy, while the other third of patients experience relapse [6]. In particular to DLBCL, the cumulative five-year survival rates are 55% due to the aggressive behaviors of these lymphomas. However, in one study from Japan, 114 patients had a five-year survival rate of 25% if the nasal cavity was the primary site, as compared to 85% if the paranasal sinuses were the primary site, and 88% of the patients had the DLBCL subtype [7]. This study patient did not have distant spread and had a favorably low stage of disease, which contributed to a favorable response with chemotherapy. If chemotherapy did not resolve the disease, then other treatment options would include radiotherapy (e.g., the distant spread of disease) and surgery (e.g., cases with life-threatening complications such as obstruction of the upper respiratory system) [8]. Surgical intervention would typically involve the decompression of the nasal passage, the opening of the paranasal sinuses, and relieving pressure in the orbits.

## Conclusions

This case demonstrates an atypical presentation of DLBCL at the nasal septum with no constitutional B symptoms (e.g., fever, weight loss, night sweats). The diagnosis was found to be DLBCL on biopsy, which was further supported by findings on CT of the sinuses and FISH. The patient’s early detection of the lymphoma was essential in prognosis, yielding a favorable stage of the disease and treatment response. Although most NHLs involve the nasal sinuses and Waldeyer’s ring, this case illustrates the importance of possible extranodal involvement of DLBCL in the nasal septum. It is essential for clinicians to be familiar with head and neck manifestations of lymphomas, especially rapidly growing subtypes such as DLBCL.
